# AtIRE1C, an unconventional isoform of the UPR master regulator AtIRE1, is functionally associated with AtIRE1B in Arabidopsis gametogenesis

**DOI:** 10.1002/pld3.187

**Published:** 2019-11-28

**Authors:** Yunting Pu, Cristina Ruberti, Evan R. Angelos, Federica Brandizzi

**Affiliations:** ^1^ MSU‐DOE Plant Research Lab Michigan State University East Lansing Michigan; ^2^ Department of Plant Biology Michigan State University East Lansing Michigan

**Keywords:** *Arabidopsis thaliana*, ER stress, gametogenesis, inositol requiring enzyme‐1, unfolded protein response

## Abstract

The unfolded protein response (UPR), a highly conserved set of eukaryotic intracellular signaling cascades, controls the homeostasis of the endoplasmic reticulum (ER) in normal physiological growth and situations causing accumulation of potentially toxic levels of misfolded proteins in the ER, a condition known as ER stress. During evolution, eukaryotic lineages have acquired multiple UPR effectors, which have increased the pliability of cytoprotective responses to physiological and environmental stresses. The ER‐associated protein kinase and ribonuclease IRE1 is a UPR effector that is conserved from yeast to metazoans and plants. IRE1 assumes dispensable roles in growth in yeast but it is essential in mammals and plants. The Arabidopsis genome encodes two isoforms of IRE1, IRE1A and IRE1B, whose protein functional domains are conserved across eukaryotes. Here, we describe the identification of a third Arabidopsis IRE1 isoform, IRE1C. This protein lacks the ER lumenal domain that has been implicated in sensing ER stress in the IRE1 isoforms known to date. Through functional analyses, we demonstrate that IRE1C is not essential in growth and stress responses when deleted from the genome singularly or in combination with an *IRE1A* knockout allele. However, we found that IRE1C exerts an essential role in gametogenesis when IRE1B is also depleted. Our results identify a novel, plant‐specific IRE1 isoform and highlight that at least the control of gametogenesis in Arabidopsis requires an unexpected functional coordination of IRE1C and IRE1B. More broadly, our findings support the existence of a functional form of IRE1 that is required for development despite the remarkable absence of a protein domain that is critical for the function of other known IRE1 isoforms.

## 1 INTRODUCTION

Environmental and physiological situations that increase the cell's secretory activity alter the homeostasis of protein synthesis in the endoplasmic reticulum (ER) and ignite a potentially lethal condition, known as ER stress. In response to ER stress, cells initiate the unfolded protein response (UPR), a set of integrated signaling pathways designed to restore ER homeostasis and growth. If the UPR fails, cells initiate irreversible death processes most likely to avoid the production and release of potentially harmful misfolded proteins (Walter & Ron, 2011).

In multicellular eukaryotes, the UPR signaling relies on multiple sensors. In vertebrates, three ER stress sensors and transducers have been identified: the protein kinase RNA‐like endoplasmic reticulum kinase (PERK) (Harding, Zhang, Bertolotti, Zeng, & Ron, 2000), the inositol requiring enzyme‐1 (IRE1) (Shamu & Walter, 1996), and the membrane‐tethered activating transcription factor (TF) ATF6 (Haze, Yoshida, Yanagi, Yura, & Mori, 1999). IRE1, ATF6, and PERK are all proteins anchored to the ER membrane via a single‐spanning transmembrane region but their signaling mechanisms are markedly different. Activated PERK controls the selective translation of the UPR TF ATF4 upon phosphorylation of the translation initiation factor eIF2‐α (Shi et al., 1998). Upon ER stress, ATF6 is released from the ER lumenal chaperone binding protein (BiP) and translocated to the Golgi where intramembrane proteolysis releases a cytosolic TF domain. This TF is then translocated to the nucleus to control UPR gene expression (Shen, Chen, Hendershot, & Prywes, 2002). IRE1 mediates the splicing of an intron in the mRNA of the UPR basic leucine zipper (bZIP)‐transcription factor Xbp1. The spliced mRNA is ligated and translated into an active TF, which is then translocated into the nucleus where it modulates the expression of UPR target genes (Yoshida, Matsui, Yamamoto, Okada, & Mori, 2001). In plants, a functional equivalent of PERK has not been identified; however, the Arabidopsis genome encodes functional homologs of the vertebrate IRE1 and ATF6, which are referred to as AtIRE1 and bZIP28, respectively (Gao, Brandizzi, Benning, & Larkin, 2008; Ruberti & Brandizzi, 2018; Ruberti, Kim, Stefano, & Brandizzi, 2015). In ER stress conditions, AtIRE1 splices the mRNA of the bZIP‐TF bZIP60 (Deng, Srivastava, & Howell, 2013; Nagashima et al., 2011), the functional homolog of Xbp1. By encoding exclusively Ire1p, which splices the mRNA of the bZIP‐TF Hac1, the yeast UPR has only one signaling branch to respond to ER stress (Shamu & Walter, 1996).

Inositol requiring enzyme‐1 is a type I membrane protein with distinct functional domains: an N‐terminal ER lumenal domain, a transmembrane domain, and a C‐terminal cytosolic domain. The understanding of the mechanisms of activation of IRE1 in non‐plant species is rather mature. The N‐terminal lumenal domain interacts with other proteins, such as BiP and unfolded peptides. The sequential dissociation of BiP and interaction of misfolded proteins with the lumenal domain of IRE1 is at the basis of IRE1 activation (Gardner & Walter, 2011; Kimata et al., 2007; Kimata, Oikawa, Shimizu, Ishiwata‐Kimata, & Kohno, 2004; Oikawa, Kimata, & Kohno, 2007). The cytosolic region of IRE1 executes endoribonuclease and kinase activity. Accumulation of misfolded proteins in the ER leads to auto‐phosphorylation of IRE1 and consequently to activation of the ribonuclease domain, which is necessary for the splicing of the target bZIP‐TF. Stresses connected to aberrancy of membrane homeostasis can activate IRE1 independently from its interaction with misfolded proteins, supporting that IRE1 may be responsive to stress in manners that are uncoupled from the lumenal domain. Indeed, a role for the transmembrane domain of IRE1 and proximal amphipathic regions has been proposed in sensing changes of ER membrane homeostasis during proteotoxic stress (Halbleib et al., 2017; Volmer, van der Ploeg, & Ron, 2013). In plants, the mechanisms of activation of IRE1 have not been defined yet, but, based on the level of functional conservation of IRE1 and of the protein domains across kingdoms, it is likely that the lumenal and transmembrane domains of IRE1 have a bearing on AtIRE1 signaling in the plant UPR as well.

Inositol requiring enzyme‐1 assumes roles that are independent from the gene‐regulatory functions of the target UPR bZIP‐TF, as supported by the evidence that IRE1‐ribonuclease activity controls the selective degradation of cytosolic mRNA transcripts, via a process known as Regulated IRE1‐Dependent Decay (RIDD) in yeast, metazoans, and plants (Hollien et al., 2009; Kimmig et al., 2012; Mishiba et al., 2013), possibly to reduce the secretory protein load in conditions of ER stress. In addition, it has been demonstrated that the cytosolic region of vertebrate IRE1 serves as a scaffold to anchor other proteins that participate in cell fate decisions (Woehlbier & Hetz, 2011). The existence of bZIP‐dependent and independent signaling supports a role of IRE1 as a critical hub integrating pro‐survival and pro‐death pathways in growth and stress.

Although IRE1 is the only ER stress sensor conserved across the genomes of yeast, vertebrates, and plants (Mori, 2009), yeast expresses only one IRE1 isoform (Shamu & Walter, 1996). However, in multicellular eukaryotes, the number of IRE1 isoforms has expanded. In the course of evolution, this has likely provided greater flexibility to accommodate tissue and cell‐specific demands of protein synthesis in stress and growth compared with single‐cell systems such as yeast. Indeed, vertebrates and plants such as *Arabidopsis thaliana* express two IRE1 isoforms (Ire1α and Ire1β in vertebrates; AtIRE1A and AtIRE1B in *A. thaliana*). In metazoans, contrarily to the loss of Ire1β, the loss of Ire1α is lethal due to placenta failure (Iwawaki, Akai, & Kohno, 2010). In *A. thaliana*, contrarily to the loss of AtIRE1A, the complete loss of AtIRE1B is lethal (Lu & Christopher, 2008); in rice, which expresses only one IRE1 isoform (OsIRE1), a loss‐of‐function mutant of OsIRE1 is lethal (Wakasa, Hayashi, Ozawa, & Takaiwa, 2012). The lethality of Ire1α, AtIRE1B, and OsIRE1 complete loss‐of‐function alleles indicates that in multicellular organisms IRE1 assumes essential roles in physiological conditions of growth in the absence of induced ER stress.

While the splicing activity of AtIRE1, which is necessary to activate bZIP60 and perform RIDD activities, has been demonstrated in plants (Mishiba et al., 2013), the mechanisms adopted by AtIRE1 to ensure cell homeostasis in physiological growth are unknown. Studies based on the phenotypical characterization of an Arabidopsis double mutant bearing a knockout allele of AtIRE1A and an AtIRE1B functional knockdown (*atire1*; (Chen & Brandizzi, 2012; Deng et al., 2013)) have reported a compromised root growth compared with wild type, supporting that AtIRE1A and AtIRE1B share at least partially overlapping roles in physiological growth. The evidence that a bZIP60 loss‐of‐function mutant is viable and exhibits no defects in physiological growth underlies that the essential role of AtIRE1B as well as the overlapping roles of AtIRE1A and AtIRE1B in organ growth are executed independently from bZIP60 (Chen & Brandizzi, 2012; Deng et al., 2013)).

**Figure 1:**
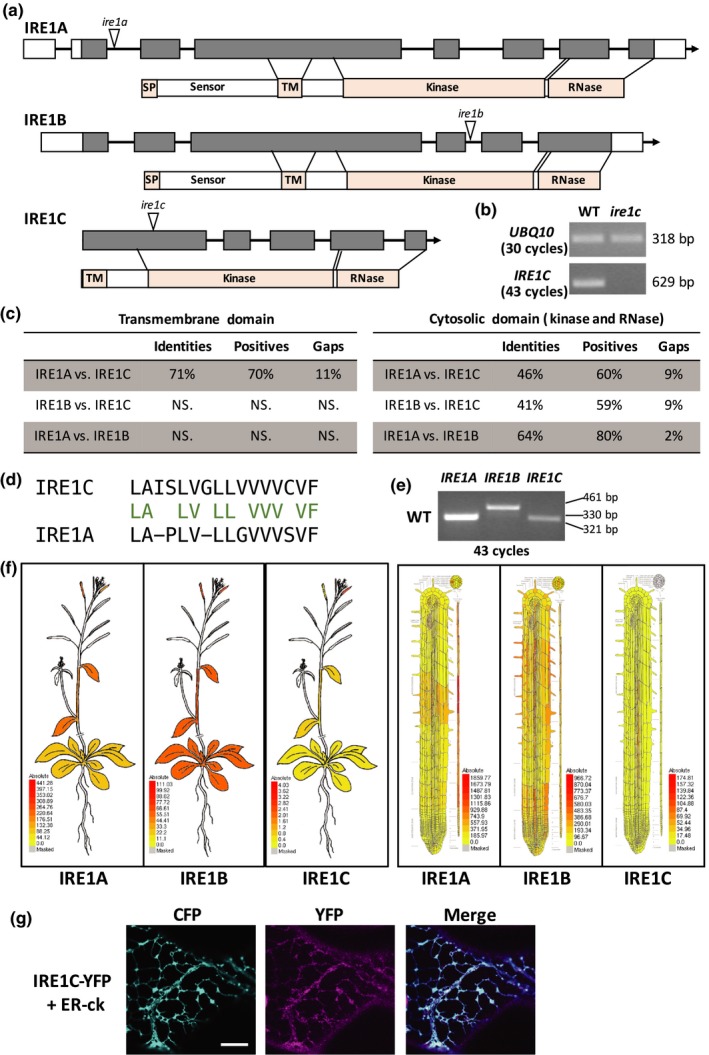
IRE1C is a sequence variant of IRE1. (a) Schematic maps of *IRE1A*, *IRE1B,* and *IRE1C* genes and proteins. Exons: gray rectangles; introns: lines. T‐DNA insertion sites in the mutant alleles are indicated by a white arrowhead in each gene. SP, signal peptide; TM, transmembrane domain. (b) *ire1c* is a knockout allele of *IRE1C*. Partial transcripts of *IRE1C* in WT and *ire1c* cDNAs were amplified with 43 cycles using RT‐PCR. *UBQ10* was used as internal control (30 PCR cycles). (c) Amino acid identity levels (%) between IRE1A, IRE1B, or IRE1C proteins through standard BLAST‐P analysis. NS., not significant. (d) Alignment of the amino acid sequences of IRE1A and IRE1C transmembrane domains. Conserved amino acids are labeled green. (e) Gene expression of *IRE1A*, *IRE1B,* and *IRE1C* in WT. Partial transcripts of *IRE1A*, *IRE1B,* and *IRE1C* genes were amplified from WT cDNA of whole seedlings with 43 cycles in PCR. (f) eFP maps of IRE1A, IRE1B, and IRE1C in whole plant and roots (Winter et al., 2007) showing expression pattern and levels of each genes. Colors indicate expression levels relative to the absolute values. (g) Representative images of transient expression of ER‐ck, IRE1C‐YFP or both constructs in tobacco leaves. Scale bar = 10 μm

Although IRE1 is conserved from yeast to plants and metazoans, its relevance to physiological growth varies across taxa. For example, IRE1 is dispensable in *Caenorhabditis elegans* (Shen et al., 2001) but essential in *Drosophila meloganaster* (Plongthongkum, Kullawong, Panyim, & Tirasophon, 2007), similar to mammals and plants, supporting the hypothesis that while the enzymatic activity of the IRE1 isoforms is conserved across eukaryotes, the degree of relevance of the IRE1 signaling in growth varies greatly. Such degree of functional relevance has possibly evolved to suit taxa‐specific features during organismal development. In this work, we aimed at gaining insights into the regulation of the plant UPR and discover genes that influence the function of AtIRE1. We report on the identification of a modified AtIRE1 protein, hereby named AtIRE1C, which lacks the ER lumenal domain but retains the transmembrane, ribonuclease, and kinase domains of the other two AtIRE1 isoforms (Figure 1a). Notably, similarly to an AtIRE1A knockout and an AtIRE1B functional knockdown, an AtIRE1C knockout is not essential for growth in physiological conditions and for the UPR in induced ER stress. However, AtIRE1C is absolutely required for plant growth when AtIRE1B function is compromised. Therefore, our results report on a unique IRE1 isoform in eukaryotes that contributes to the UPR and highlight a plant‐specific innovation of UPR management in growth.

## 2 EXPERIMENTAL PROCEDURES

### 2.1 Plant materials, growth conditions, and allele transmission analyses

Arabidopsis Columbia‐0 (Col‐0) was used as wild‐type (WT) reference in this study. T‐DNA insertion lines of *AtIRE1A* (here dubbed *ire1a* (WiscDsLox420D09) (Chen & Brandizzi, 2012)), *AtIRE1B* (here dubbed *ire1b* (SAIL_238_F07) (Chen & Brandizzi, 2012; Deng et al., 2011)), and *AtIRE1C* (here dubbed *ire1c* (SALK_204405)) were obtained from the Arabidopsis Biological Resource Center. Seeds were sterilized with 30% (v/v) bleach and 0.1% (v/v) Triton X‐100 (Sigma) for 20 min, followed by five washes of 5 min each with sterile water. Sterilized seeds were stored at 4°C in darkness for at least 2 days to allow synchronization and plated on half‐strength Linsmaier and Skoog (LS) medium (2.36 g/L LS basal medium [Caisson], 1% [w/v] sucrose, and 1.2% [w/v] agar). Seedlings were grown vertically in a controlled growth chamber at 21°C under long‐day conditions (16‐hr light) with light intensity of 110 μE m^−2^ s^−1^. For quantitative real‐time polymerase chain reaction (PCR) analysis of gene expression in WT and *ire1a ire1b* mutant, Arabidopsis seeds were grown on half‐strength LS medium with 2% sucrose for 7 days, and root tissues were harvested for RNA extraction. For the allele transmission analyses, pollen grains from freshly dehiscent flowers from plants with the indicated genotypes in Table 1 were deposited on the stigma of emasculated flowers. Seeds from the siliques originated from the crosses where germinated after surface sterilization on solid LS medium as described above. The genomic DNA from the germinated seedlings was extracted and subjected to PCR amplification to identify the presence of the T‐DNA, as detailed below in the DNA and mRNA analyses section. Significance levels in the differences in allele transmission were estimated using chi‐square test.

**Table 1:** Transmission of the *ire1c* mutant allele along with *ire1a* or *ire1b* alleles

(a) Parental genotype		Progeny genotype					Total	*p*‐value
Female ♀	Male ♂							
AaCc	AACC		AACC	AaCC	AACc	AaCc		
		Expected ratio	1	1	1	1	54	NS.
		# Observed	14	13	12	15		
BbCc	BBCC		BBCC	BbCC	BBCc	BbCc		
		Expected ratio	1	1	1	1	111	<.001
		Observed	0	61	50	0		
AACC	AaCc		AACC	AaCC	AACc	AaCc		
		Expected ratio	1	1	1	1	58	NS.
		Observed	13	16	14	15		
BBCC	BbCc		BBCC	BbCC	BBCc	BbCc		
		Expected ratio	1	1	1	1	71	<.001
		Observed	1	30	40	0		

(b) Selfing genotype		Progeny genotype									Total	*p*‐value
AaCc		AACC	AACc	AAcc	AaCC	AaCc	Aacc	aaCC	aaCc	aacc		
	Expected ratio	1	2	1	2	4	2	1	2	1		
	# Observed	6	7	4	6	10	7	5	7	5	57	NS.
BbCc		BBCC	BBCc	BBcc	BbCC	BbCc	Bbcc	bbCC	bbCc	bbcc		
	Expected ratio	1	2	1	2	4	2	1	2	1		
	# Observed	0	0	16	0	28	0	17	0	0	61	<.001

Note

(a–b) Reciprocal crosses between heterozygous *ire1a ire1c (AaCc)* mutant or heterozygous *ire1b ire1c (BbCc)* mutant and WT (a), and their self‐pollination (b) showing abnormal segregation of *ire1c* along with *ire1b*. *p*‐value of the observed segregation ratio compared with expected Mendelian segregation ratio is calculated with chi‐square test. *p*‐value < .05 is considered as significant.

Abbreviation NS., not significant.

### 2.2 Stress treatment and phenotypic analyses

For chronic ER stress treatment (Chen & Brandizzi, 2012; Deng et al., 2013; Martínez & Chrispeels, 2003; Nagashima et al., 2011), Arabidopsis seeds were germinated on solid half‐strength LS medium containing 25 nM tunicamycin (Tm, Sigma‐Aldrich) or same volume of DMSO (Tm solvent; mock control) and grown for 10 days. The root length was measured from at least 54 seedlings. Fresh weight of whole seedlings, root, or shoot tissue of 15 seedlings was measured. For recovery assays from temporary ER stress (Ruberti & Brandizzi, 2018), 5‐day‐old seedlings grown on solid half‐strength LS medium were transferred to liquid medium containing 0.5 μg/ml Tm for 6 hr, followed by transfer to regular solid half‐strength LS medium for 4 days. Root length of 50 seedlings was measured. Fresh weight of whole seedlings or shoot tissue of 15 seedlings was measured. The relative root length and the relative fresh weight were calculated as described earlier (Meng, Ruberti, Gong, & Brandizzi, 2017), using the measurement value in Tm treatment divided by the value in DMSO treatment. Statistical analyses were performed by Student's two‐tailed *t* test, with a *p*‐value < .05 considered as significant.

### 2.3 Phenotypic evaluation of siliques and pollen viability assay

The siliques at the 6th–10th positions of the primary flower shoots were removed, and the average silique length and thickness were measured using ImageJ. The average seed number per silique and percentage of aborted seeds per silique were calculated after opening each silique and observed with a dissecting microscope. For pollen viability assay, pollen grains in anthers of freshly dehiscent WT or mutant flowers were stained with Alexander's stain as described earlier (Alexander, 1969) and analyzed using the Zeiss Axio Imager M2 microscope (Zeiss). Percentage of aborted pollen was calculated on 100 pollen grains pooled from flowers of each genotype in 3 independent biological replicates. Percentage of aborted pollen was calculated on 100 pollen grains pooled from flowers of each genotype in 3 independent biological replicates.

### 2.4 Plasmid construction

*IRE1C* genomic DNA fragment was amplified by PCR with primers in Table S1 using Phusion High‐Fidelity DNA polymerase (New England Biolabs Inc.). The PCR product was cloned into the pCR8 vector using the PCR8/GW/TOPO‐TA cloning kit (Thermo Fisher) and then into the Gateway compatible destination vector pEarlyGate 101 (Earley et al., 2006) to generate the 35S::IRE1C‐YFP construct.

### 2.5 Subcellular localization assay and confocal microscopy

*Agrobacterium tumefaciens* (GV3101) containing the 35S::IRE1C‐YFP or ER‐ck (Nelson, Cai, & Nebenführ, 2007) vector was suspended to OD_600_ = 0.05 and infiltrated into leaves of 3‐week‐old *Nicotiana benthamiana* allowing expression for 2–3 days. Leaf epidermal cells were observed by an inverted laser‐scanning confocal microscope (Nikon A1RSi). CFP fluorescence was monitored at excitation wavelength of 440 nm and with a bandpass of 467–502 nm emission filter, and YFP fluorescence was monitored at excitation wavelength of 514 nm and with a bandpass of 570–620 nm emission filter. Images were analyzed using ImageJ software (Schneider, Rasband, & Eliceiri, 2012).

### 2.6 DNA and mRNA analyses

Genomic DNA was extracted using Edwards’ method (Edwards, Johnstone, & Thompson, 1991). The presence of the T‐DNA insertion was analyzed using the primers listed in Table S1. Total RNA was extracted using the NucleoSpin RNA Plant kit (Macherey‐Nagel) and was reverse‐transcribed to cDNA (iScript cDNA synthesis kit, Bio‐Rad) for RT‐PCR or quantitative real‐time PCR (qRT‐PCR). For RT‐PCR, cDNA was used for amplification with specific primers (Table S1) using GoTaq polymerase (Promega). For qRT‐PCR, gene expression was detected using CYBR green (Fast SYBR Green Master Mix, Thermo Fisher) through the Applied Biosystems 7500 fast real‐time PCR system, and data were analyzed using ∆∆C_T_ method with *UBQ10* or *ACT2* as internal control as indicated. Each biological replicate contains three technical replicates, and values are averages from three independent experiments. Statistical analysis was established with Student's two‐tailed *t* test with *p*‐value < .05 considered as significant.

## 3 RESULTS

### 3.1 IRE1C is an unconventional isoform of IRE1 in Arabidopsis

The protein domains of the two Arabidopsis isoforms of AtIRE1, AtIRE1A and AtIRE1B (here dubbed IRE1A and IRE1B, respectively), have been identified (Koizumi et al., 2001). Both isoforms have a ER stress sensing (sensor) domain localized in the ER lumen, an ER transmembrane domain (TM), a kinase domain, and a ribonuclease domain (Figure 1a and Figure S1). The presence of two isoforms in *A. thaliana* but only one isoform in rice prompted us the hypothesis that the *A. thaliana* isoforms could be the product of gene duplication. Therefore, we queried the *A. thaliana* genome to explore the possibility that additional isoforms could exist. Using BLAST‐P analyses, we identified an isoform of AtIRE1 that we named *AtIRE1C* (*AT3G11870*; here dubbed *IRE1C*). This isoform was also mentioned as an IRE1‐like gene in a previous work but was not characterized (Deng et al., 2011). The primary sequence of IRE1C contains 554 amino acids. Membrane topology analyses indicated the presence of a putative signal peptide at the N‐terminus and a TM domain close to the N‐terminus (Bernsel et al., 2008). Compared with IRE1A and IRE1B, IRE1C exhibits 71% identity at the TM domain (Figure 1c,d and Figure S1). Strong amino acid identity was also verified for the cytosolic domain, which encompasses the kinase and ribonuclease domains, with a 46% and 41% identity compared with IRE1A and IRE1B, respectively (Figure 1c and Figure S1). These bioinformatics analyses suggest that IRE1C is an isoform of IRE1A and IRE1B. Surprisingly however, the sequence of IRE1C lacks the extensive ER lumenal (Figure 1a, Figure S1), which in non‐plant species functions as a sensor of unfolded or misfolded proteins in the ER.

We next tested whether *IRE1C* is expressed. To do so, we carried out RT‐PCR analyses with specific primer sets (Table S1) and identified the presence of transcripts in 5‐day‐old whole seedlings grown in standard conditions of growth (Figure 1e). Both *IRE1A* and *IRE1B* exhibit a low level of expression (Chen & Brandizzi, 2012), and therefore, 43 PCR cycles were used to ensure detection of *IRE1* expression. When compared to *IRE1A* and *IRE1B*, *IRE1C* showed much lower expression in the whole plant (Figure 1e), as reflected in eFP gene expression browser analyses (Winter et al., 2007) (Figure 1f). These results indicate that *IRE1C* is a functional gene, at least in terms of expression.

We next sought to establish the subcellular localization of the IRE1C protein. The high identity of the TM region of the IRE1A and IRE1C proteins (Figure 1c,d) suggested that IRE1C could be localized to the ER. To test this possibility, we fused the C‐terminus of IRE1C to a yellow fluorescent protein (YFP) for subcellular localization analyses and transiently expressed it in tobacco leaf epidermal cells (Figure 1g). When expressed alone, IRE1C‐YFP was localized to a network‐like structure that is typical of the ER (Figure S1). The ER localization was confirmed in cells co‐expressing the ER lumenal marker ER‐ck, which is based on a cyan fluorescent protein targeted to the ER (Nelson et al., 2007). We found overlap of the YFP and CFP fluorescent signals (Figure 1g, Figure S2). Therefore, at least in the system adopted in our analyses, IRE1C‐YFP is localized to the ER.

### 3.2 Loss of *IRE1C* when *IRE1B* is compromised leads to defects in gametogenesis

Having established that *IRE1C* is expressed (Figure 1e), we next aimed to test its relevance in plant growth and development. A complete loss‐of‐function allele of *IRE1A* (*ire1a*) is viable and does not show obvious growth phenotypes compared with wild type (WT) (Chen & Brandizzi, 2012). In net contrast, a complete loss of function of *IRE1B* is lethal (Lu & Christopher, 2008). A functional knockdown of *IRE1B* is viable and, similar to *ire1a*, it does not show growth defects, supporting that IRE1A and IRE1B have largely overlapping roles in regulation of plant growth (Chen & Brandizzi, 2012; Deng et al., 2013). This conclusion was further supported by the evidence that a double *ire1a ire1b* mutant has a reduced root length phenotype compared with WT and the single *ire1a*, *ire1b* mutants (Chen & Brandizzi, 2012; Deng et al., 2013; Ruberti, Lai, & Brandizzi, 2018). To test whether IRE1C plays a role in plant growth, we isolated *ire1c*, a knockout allele of *IRE1C* (Figure 1b). Due to the low expression level of *IRE1C*, we increased the PCR cycle number to 43 cycles to ensure there was no expression of *IRE1C* in the *ire1c* mutant, while expression of UBQ10 as internal control was limited 30 PCR cycles to avoid signal saturation. We found that similar to *ire1a* and *ire1b*, *ire1c* did not show evident growth and developmental phenotypes, supporting that either IRE1C is not involved in processes related to growth or development or that it shares overlapping roles with IRE1A and/or IRE1B. To test and distinguish these possibilities, we crossed *ire1c* with *ire1a*, *ire1b*, and *ire1a ire1b*. We were able to recover *ire1a ire1c* double mutants, which did not exhibit evident plant phenotypes. In net contrast, we were not able to isolate homozygous *ire1b ire1c* double mutant or an *ire1a ire1b ire1c* triple mutant (Table 1). These results indicate that the *ire1b ire1c* combination is lethal.

**Figure 2:**
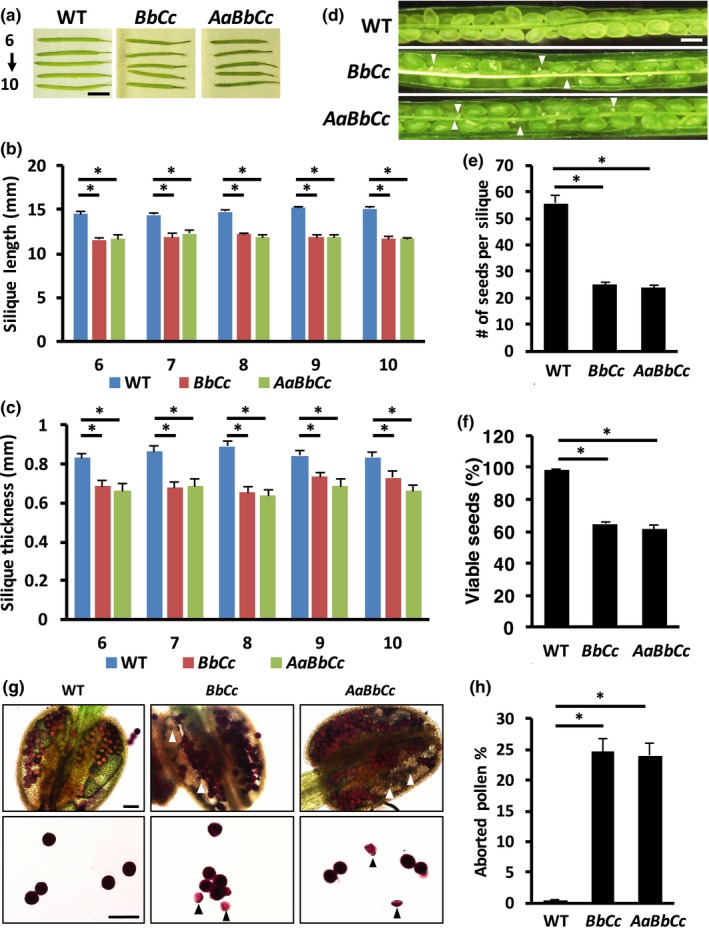
*ire1b*+/− *c*+/− *and ire1a*+/− *b*+/− *c*+/− mutants exhibits impaired seed development. (a) Representative images of the 6th to 10th siliques of WT, *ire1b*+/− *c*+/− * (BbCc),* and *ire1a*+/− *b*+/− * c*+/− * (AaBbCc)* mutants. Scale bar = 5 mm. (b–c) Measurements of the silique length (b) and silique thickness (c) of the 6th to 10th siliques of WT, *ire1b*+/− *c*+/− *(BbCc),* and *ire1a*+/− *b*+/− *c*+/−* (AaBbCc)* mutants. Error bars refer to standard error values. Asterisks indicate *p* < .05. (d) The siliques of the *ire1a*+/− *b*+/− *c*+/− showed gaps with abnormal seed development. Scale bar = 100 μm. (e–f) The number of seeds in each silique (e) and the percentage of aborted seeds in each silique (f) of WT, *ire1b*+/− *c*+/− *(BbCc),* and *ire1a*+/− *b*+/− *c*+/− *(AaBbCc)* mutants. Error bars refer to standard error values. Asterisks indicate *p* < .05. (g) Alexander's staining of the pollen of WT, *ire1b*+/− *c*+/− *(BbCc),* and *ire1a*+/− *b*+/− *c*+/− *(AaBbCc)* mutants. Viable pollen grains are stained purple; aborted pollen grains are pink and shrunk, and are indicated by arrowheads. Scale bar = 50 μm

We next aimed to gain insights into the apparent lethality of the *ire1b ire1c* double mutant. We first tested whether seed development was impaired in the mutant. Therefore, we inspected the siliques of self‐pollinated *ire1b*+/− *ire1c*+*/-* and *ire1a*+/− *ire1b*+/− *ire1c*+/− mutants (note that + and − indicate WT allele and loss‐of‐function allele, respectively). Both the length and thickness of the *ire1b*+/− *ire1c*+*/-* or *ire1a*+/− *ire1b*+/− *ire1c*+/− mutant siliques were reduced compared with WT (Figure 2a‐c). These findings prompted the hypothesis that *IRE1C* could be involved in seed development. This was supported by further inspection of the WT, *ire1b*+/− *ire1c*+/−, and *ire1a*+/− *ire1b*+/− *ire1c*+/− mutant siliques, which revealed a reduced seed set and apparently unfertilized ovules or failed fertilized ovules in the mutant background (Figure 2d‐f). These results suggested both male and female gametophyte defects in the mutant. To test this hypothesis, we analyzed pollen viability with Alexander's stain (Figure 2g‐h). An abundance of purple stained pollen grains was observed in WT indicating viable pollens. In net contrast, numerous aborted pollen grains were detected in both *ire1b*+/− *ire1c*+/− and *ire1a*+/− *ire1b*+/− *ire1c*+/− mutants. Pollen viability is normal in the *ire1a ire1b* mutant (Deng et al., 2016). Therefore, the pollen viability defects in the *ire1b*+/− *ire1c*+/− and *ire1a*+/− *ire1b*+/− *ire1c*+/− mutants are likely due to the *ire1c* allele, and *IRE1C* is likely to have a key role in male gametophyte development. To confirm a male gametophyte defect due to the *ire1c* mutation, we performed reciprocal crosses of *ire1a*+/− *ire1c*+/− with WT and *ire1b*+/− *ire1c*+/− with WT (Table 1, Figure S3). We found that segregation of *ire1a* and *ire1c* alleles in the *ire1a*+/− *ire1c*+/− mutant exhibited no difference from the expected Mendelian segregation ratio. In net contrast, the *ire1b* and *ire1c* alleles showed markedly abnormal segregation when either the WT or the mutant was used as pollen source to generate an *ire1b*+/− *ire1c*+/− mutant (Table 1a). This is significantly different from the Mendelian segregation ratio that is 1:1: 1:1 for all 4 allele combinations. Out of the 71 seedlings tested from the crosses using *ire1b*+/− *ire1c*+/− as pollen source, we found *ire1b*+/− *ire1c*+/+ and *ire1b*+/+ *ire1c*+/− in the progenies with a 0.75:1 ratio, which is not significantly different from the expected Mendelian ratio 1:1. However, no *ire1b*+/− *ire1c*+/− and only 1 WT progeny was identified, indicating that the male gametophyte is impaired when the *ire1b* and *ire1c* alleles are combined. Furthermore, out of the tested 111 seedlings from the crosses using WT as pollen source, the segregating progeny was *ire1b*+/− *ire1c*+/+ and *ire1b*+/+ *ire1c*+/− with a ratio of 1.22:1, which is also not significantly different from the expected 1:1 ratio; however, no *ire1b*+/− *ire1c*+/− or WT was detected. These results indicate that the female gametophyte is also largely impaired in the *ire1b ire1c* allelic combination. In addition, self‐pollination of *ire1a*+/− *ire1c*+/− also showed normal segregation matching the Mendelian segregation ratio; however, abnormal segregation of *ire1b* and *ire1c* was found in the self‐pollinated *ire1b*+/− *ire1c*+/− mutant (Table 1b), supporting the abnormal segregation in the *ire1b*+/− *ire1c*+/− reciprocal crosses. Taken together, these results indicate that both male gametogenesis and female gametogenesis are impaired in the *ire1b*+/− *ire1c*+/− mutant, supporting that *IRE1B* and *IRE1C* function together in gametogenesis.

The verified role of IRE1C in gametogenesis triggered us to test whether *IRE1C* exhibits different expression levels in various reproductive tissues. We therefore conducted quantitative real‐time PCR of IRE1C in the siliques and flowers (Figure S4). We found that the pistil contained about twofold higher expression than the stamen. No significant difference was detected between the complete flower and the flower without pistils or stamens, or between the complete siliques and the siliques without seeds. Interestingly, the siliques, with or without seeds, showed much higher IRE1C expression than the other tested organs, suggesting IRE1C might also play a role in the silique and seeds development.

### 3.3 IRE1C is not essential in the UPR in conditions of chronic and transient ER stress but likely provides ribonuclease activity in the UPR

**Figure 3:**
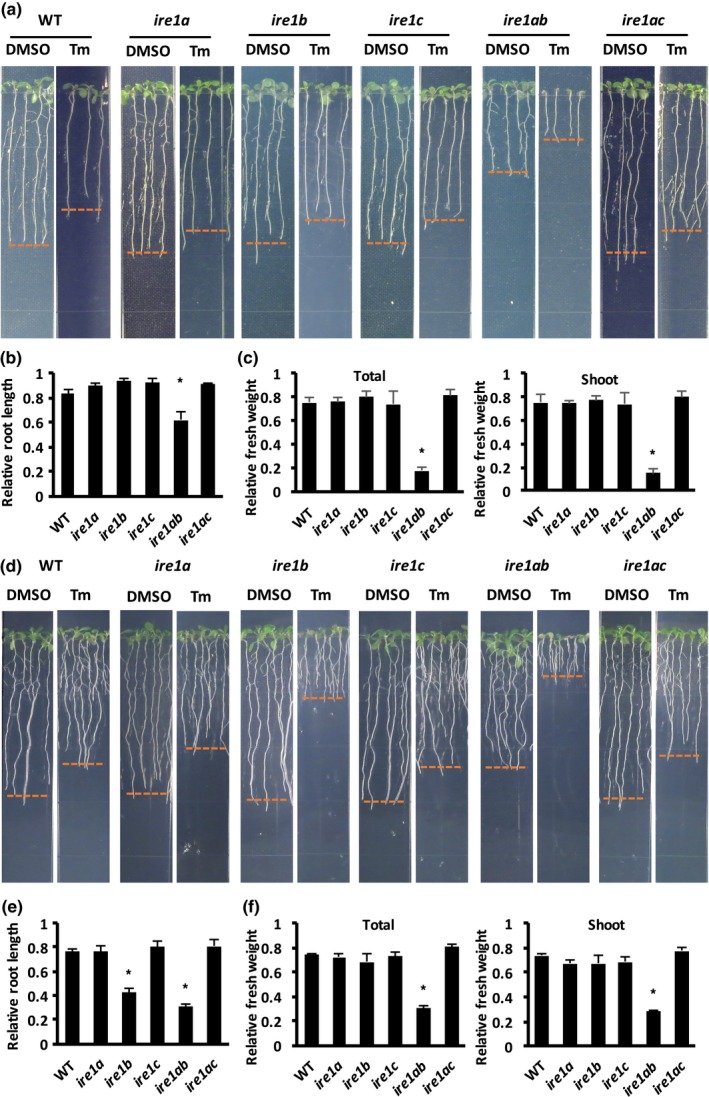
IRE1C is not essential in the ER stress‐induced UPR. (a) Representative images of seedlings of WT, *ire1a*, *ire1b*, *ire1c*, *ire1a ire1b (ire1ab),* and *ire1a ire1c (ire1ac)* genotypes upon chronic ER stress. Root length in each treatment condition (i.e., DMSO, mock control; Tm, ER stress) is indicated by red dashed lines for each genotype. (b) Representative relative root length (Tm/DMSO) of WT and the *ire1* mutants of the experiment presented in panel (a). (c) Representative relative fresh weight (Tm/DMSO) of the whole seedlings (total) or shoot tissue of WT and the *ire1* mutants of the experiment presented in panel (a). (d) Representative images of seedlings of WT, *ire1a*, *ire1b*, *ire1c*, *ire1a ire1b,* and *ire1a ire1c* mutants at 4 days of recovery from temporary ER stress. The root length for each genotype and condition is indicated by red dashed lines. (e) Representative relative root length (Tm/DMSO) of WT and the *ire1* mutants of the experiment presented in panel (d). (f) Relative fresh weight (Tm/DMSO) of the whole seedlings (total) or shoot tissue of WT and the *ire1* mutants of the experiment presented in panel (d). All bar graphs are averages from three independent experiments. Error bars refer to standard error values. Asterisks indicate *p* < .05

We next asked whether IRE1C could be involved in ER stress responses. In plants, ER stress responses are generally tested by observing the growth of seedlings exposed to prolonged treatment with ER stress‐inducing drugs, such as Tm (Chen & Brandizzi, 2012; Deng et al., 2013; Martínez & Chrispeels, 2003; Nagashima et al., 2011). In addition, ER stress responses can be assayed in conditions of recovery from a short ER stress‐inducing treatment. The latter experimental conditions allow following the IRE1‐dependent UPR at a molecular level (i.e., induction of IRE1‐bZIP60‐responsive genes such as *BiP3* as well as expression of unspliced *bZIP60*, and production of spliced *bZIP60*) (McCormack, Liu, Jordan, & Pajerowska‐Mukhtar, 2015; Mishiba et al., 2013; Ruberti et al., 2018). Both IRE1A and IRE1B have been shown to function in the UPR in ER stress conditions, such that alone each gene is dispensable in mounting effective ER stress responses; however, functional loss of both genes leads to inefficient UPR (Chen & Brandizzi, 2012). To investigate an involvement of IRE1C in ER stress responses, we assayed the phenotype of *ire1c* and *ire1a ire1c*, using WT, *ire1a*, *ire1b,* and *ire1a ire1b* as controls in chronic ER stress. Arabidopsis seeds were directly germinated on half‐strength LS medium with 25 nM Tm and grown for 10 days; the root length, fresh weight of whole seedling, and fresh weight of shoot were measured after the treatment (Figure 3a‐c). As expected (Chen & Brandizzi, 2012; Deng et al., 2013; Ruberti et al., 2018), *ire1a* and *ire1b* single mutants showed no significant differences compared with WT, while *ire1a ire1b* double mutant did not survive chronic ER stress. The *ire1c* mutant also showed similar survival and growth phenotype compared with WT. We then extended our analyses to the *ire1a ire1c* double mutant. We established that, in net contrast to the *ire1a ire1b* double mutant, the *ire1a ire1c* did not show significant differences compared with WT. These results infer that the loss of IRE1C alone does not influence pro‐survival processes in chronic ER stress.

Next, we analyzed the involvement of IRE1C in ER stress responses upon recovery from temporary ER stress. For these analyses, 5‐day‐old seedlings were treated in liquid half‐strength LS medium with 0.5 μg/ml Tm for 6 hr and then transferred to drug‐free growth solid medium for 4 days. Root length, fresh weight of whole seedling, and fresh weight of shoot were measured after the treatment (Figure 3d‐f). As reported earlier (Ruberti & Brandizzi, 2018), the *ire1a ire1b* double mutant showed severe growth defects at the end of the treatment, as supported by the significantly reduced root length and fresh weight. Although *ire1b* has no difference in total seedling or shoot fresh weight, it exhibited significantly reduced root length compared with WT. On the other hand, *ire1a* did not show differences compared with WT. These results indicated that IRE1B plays only partially overlapping roles with IRE1A in root growth in ER stress recovery conditions. We also established that both *ire1c* and *ire1a ire1c* mutants exhibited no growth defects compared with WT, suggesting that IRE1C, unlikely IRE1B, is not essential in growth recovery from temporary ER stress.

**Figure 4:**
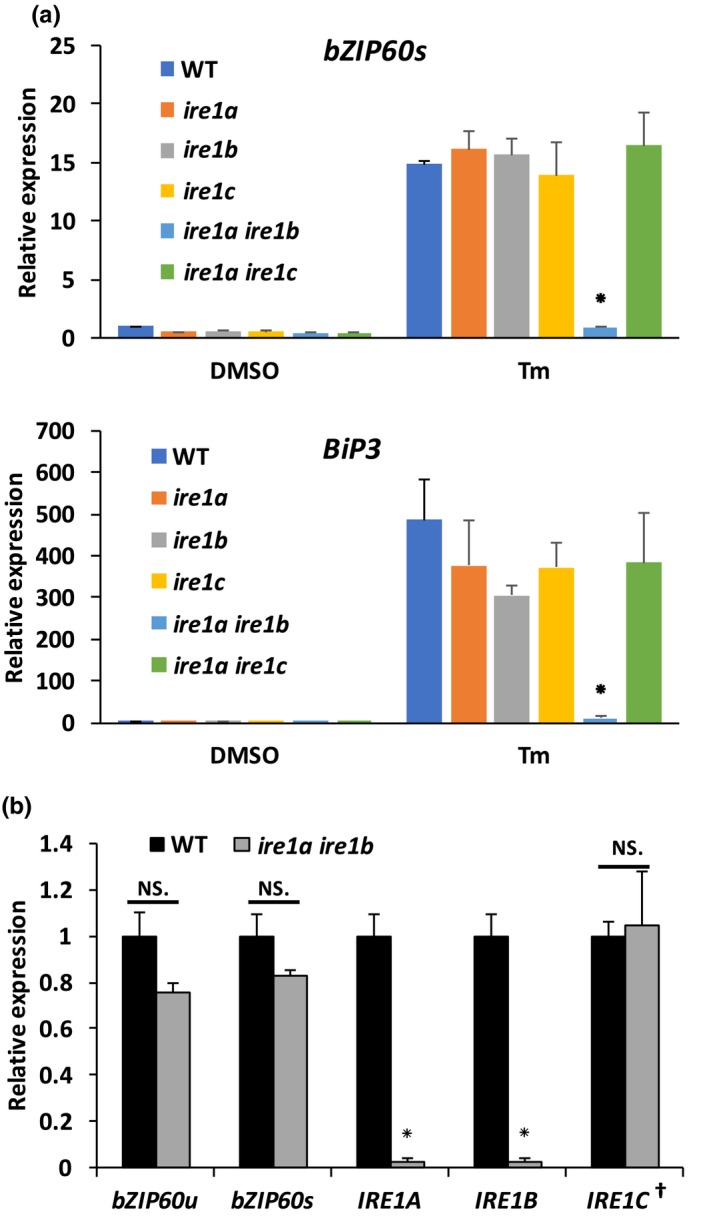
IRE1C likely exerts *bZIP60* splicing activity. (a) Quantitative real‐time PCR of spliced *bZIP60* or *BiP3* in WT and the *ire1* mutants. *bZIP60s*, spliced *bZIP60*. (b) Quantitative real‐time PCR of unspliced or spliced *bZIP60*, *IRE1A*, *IRE1B,* and *IRE1C* in WT and the *ire1a ire1b* mutant. *bZIP60u*, unspliced *bZIP60*; *bZIP60s*, spliced *bZIP60*. †, double amount of cDNA template was used for *IRE1C* expression compared with other genes to ensure reliable detection. All bar graphs are averages from three independent experiments. *UBQ10* was used as reference gene. Error bars refer to standard error values. Asterisks indicate *p* < .05. NS., not significant

We next analyzed the expression of UPR marker genes in adaptive UPR (i.e., actuation initial ER stress responses in conditions of ER stress) by focusing on spliced *bZIP60* (*bZIP60s*), and *BiP3*, which have been used to detect the IRE1‐regulated adaptive UPR signaling (Chen & Brandizzi, 2012; Martínez & Chrispeels, 2003; Nagashima et al., 2011; Ruberti et al., 2018). Spliced *bZIP60* can be detected and distinguished from unspliced *bZIP60* using specific amplification primers (Moreno et al., 2012). We used WT, *ire1a*, *ire1b*, *ire1c* single mutants, and the *ire1a ire1b* and *ire1a ire1c* double mutants. Five‐day‐old Arabidopsis seedlings were treated for 6 hr with 0.5 μg/ml Tm to induce ER stress (Ruberti et al., 2018) and then harvested for quantitative gene expression analyses using qRT‐PCR (Figure 4a). In Tm treatment conditions, all the single mutants and the *ire1a ire1c* double mutant exhibited no differences in the expression of spliced *bZIP60* or *BiP3* compared with WT; only *ire1a ire1b* showed a reduced expression level of both genes, which was similar to DMSO control treatment. These results support that, similar to IRE1A and IRE1B, IRE1C alone is not essential in the adaptive UPR signaling. Furthermore, we noticed that a residual level of *bZIP60* in the *ire1a ire1b* mutant. This was curious considering that the T‐DNA insertion of *ire1b* allele leads to a lack of transcript encoding the ribonuclease domain of the protein (Chen & Brandizzi, 2012). In alternative, we hypothesized that there could exist ribonuclease function of IRE1C in the *ire1a ire1b*. To test this, we performed qRT‐PCR to quantitatively detect unspliced and spliced *bZIP60*, *IRE1A*, *IRE1B,* and *IRE1C* expression in WT and *ire1a ire1b* mutant (Figure 4b). For these experiments, we used roots, in which all the *IRE1* isoforms are expressed (Figure 1f). Arabidopsis seeds were grown on Tm‐free growth medium, and root tissues were harvested for RNA extraction. The qRT‐PCR analyses were performed using primers targeting the last exon of each gene, which contains the genetic information for the proteins’ ribonuclease domain (Table S1), using *UBQ10* as internal control. We found that the expression levels of both unspliced and spliced forms of *bZIP60* were not significantly reduced in *ire1a ire1b* and WT, as verified earlier (Figure 4a; DMSO). *IRE1C* also showed similar expression levels in the *ire1a ire1b* mutant compared with WT, supporting that the loss of the other IRE1 isoforms does not induce compensatory changes in *IRE1C* expression. Nonetheless, similar to the *IRE1A* knockout, *IRE1B* had extremely reduced expression to nearly no expression. These results support earlier findings that the transcripts encoding the ribonuclease domain of IRE1B are unlikely present in the *ire1a ire1b* mutant (Chen & Brandizzi, 2012) and imply that the basal level of *bZIP60* splicing verified in this background is likely due to IRE1C.

## 4 DISCUSSION

The key role of IRE1 in actuating ER stress responses is conserved in yeast, mammals, and plants (Mori, 2009). However, the relevance of IRE1 to organismal survival varies immensely across eukaryotes. For example, in yeast there is only one isoform of *IRE1*, *Ire1p* (Shamu & Walter, 1996), which is dispensable for viability (Cox, Shamu, & Walter, 1993). Animals have two isoforms, *Ire1α* and *Ire1β*, and the loss of *Ire1α* leads to lethality in mammals (Iwawaki, Akai, Yamanaka, & Kohno, 2009). In plants, IRE1 exhibits variation in number and relevance to the life of the organism and this appears to be linked to the number of IRE1 isoforms. In rice, there is only one IRE1 isoform and its loss is lethal (Wakasa et al., 2012). Previous to our work, only two IRE1 homologs in Arabidopsis had been identified and studied, IRE1A and IRE1B (Koizumi et al., 2001). In addition, the null allele *ire1b* is lethal (Lu & Christopher, 2008). Our study has revealed the existence of a modified form of IRE1 in Arabidopsis, IRE1C, which has high identity of the transmembrane domain, kinase domain, and ribonuclease domain with IRE1A and IRE1B, but lacks the sensor domain that is present in all the other IRE1 isoforms known to date. We demonstrated that IRE1C is necessary for gametogenesis, supporting a biological role for this IRE1 isoform at least in organismal development.

Among eukaryotes, IRE1C is found in *A. thaliana* and *Arabidopsis lyrata* (Figure S5), and a potential IRE1C homolog protein is identified in *Capsella rubella*, which also belongs to the Brassicaceae family as *A. thaliana* and *A. lyrata*. This suggests that IRE1C might be originated from partial gene duplication and might be a plant‐specific IRE1 homolog. The transmembrane domains of IRE1A and IRE1C show a high degree of identity at amino acid level. In net contrast, compared with IRE1A and IRE1C, the transmembrane domain of IRE1B shows a marked divergence, supporting the hypothesis that *IRE1C* may have originated from a recent, partial *IRE1A* duplication.

Like the non‐plant IRE1 proteins, IRE1A and IRE1B have a typical type I protein structure with an N‐terminal signal peptide and a putative transmembrane domain (i.e., stop‐transfer sequence) as an ER membrane anchor. The high identity of transmembrane domain of IRE1C and IRE1A suggested to us that IRE1C could also localize at the ER. Through confocal microscopy analyses on the subcellular distribution of IRE1C, we established that IRE1C is indeed localized at the ER, as supported by co‐localization with an ER marker. The lack of accumulation of IRE1C to the apoplast, as it would be expected for a secretory protein with a N‐terminal signal peptide and no ER retention signal, argues in favor of a role of the IRE1C signal peptide as an ER targeting sequence and membrane anchor. Under this light, IRE1C assumes the topology of a type III membrane protein, similar to most members of the cytochrome P‐450 family for example (Goder & Spiess, 2001), where the N‐terminal region serves as a targeting signal and a transmembrane anchor, and only a few amino acid residues are exposed in the ER lumen.

Both IRE1A and IRE1B have been shown to be critical for the UPR activation in ER stress conditions (Chen & Brandizzi, 2012). Although our data show that IRE1C is localized to the ER, the absence of a lumenal sensor domain, which is present in the other IRE1 isoforms, raises the question on whether IRE1C may function in the UPR trigged by ER stress. The remaining low levels of *bZIP60* mRNA splicing in the *ire1a ire1b* mutant in both this study and previous work on the UPR (Angelos & Brandizzi, 2018; Chen & Brandizzi, 2012; Gaguancela et al., 2016) prompted us to test whether this may be due to IRE1C. Our data showed that both IRE1A and IRE1B had nearly no ribonuclease domain expressed in the *ire1a ire1b* mutant and therefore support the hypothesis that IRE1C may have ribonuclease activity. In alternative, other proteins may exert non‐degradative IRE1‐like ribonuclease activity toward *bZIP60* mRNA. While this possibility cannot be excluded, the recognition site of the hairpin‐loop in the bZIP mRNA is stringent for IRE1 (Cox & Walter, 1996; Gonzalez, Sidrauski, Dorfler, & Walter, 1999; Yoshida et al., 2001). This consideration and the high identity of the ribonuclease domain of IRE1C with the other Arabidopsis IRE1 isoforms pose that *bZIP60* mRNA is unlikely spliced by proteins other than IRE1C in the *ire1a ire1b* mutant.

We further tested the function of IRE1C in the UPR triggered in conditions of chronic ER stress or in ER stress recovery. The *ire1c* and *ire1a ire1c* mutants showed no obvious plant phenotype in either experimental conditions. Similarly, we found no difference in *bZIP60* splicing levels and *BiP3* expression in either the *ire1c* or *ire1a ire1c* mutant under these conditions compared with WT. Nonetheless, we found that the *ire1a ire1b* mutant showed marked plant phenotypes and failed to activate the UPR to WT levels, as also previously reported (Chen & Brandizzi, 2012; Deng et al., 2013; Ruberti et al., 2018). Our findings indicate that IRE1C alone and together with IRE1A is dispensable in ER stress‐induced UPR. Since it was not possible to isolate an *ire1b ire1c* mutant, whether IRE1C shares any roles with IRE1B in ER stress‐induced UPR with IRE1B is yet to be determined. Our data show that the *ire1a ire1b* mutant exhibited growth defects in both chronic ER stress and temporary ER stress recovery conditions. Furthermore, we found that the *ire1b* mutant has root elongation defects in recovery from temporary ER stress, suggesting that IRE1B is prominent in root growth in conditions of temporary ER stress recovery. Therefore, the UPR may be differently controlled by the IRE1 isoforms in the various plant tissues. Thus, although IRE1C showed no apparent role in root development in ER stress resolution, it may have functions in the regulation of ER stress‐induced UPR in other tissues.

Studies in animals and plants have shown that IRE1 executes essential functions in physiological growth (Bao, Bassham, & Howell, 2019; Chen & Brandizzi, 2013; Coelho & Domingos, 2014; Deng et al., 2016). The knockout alleles of the mammalian *Ire1α*, the Arabidopsis *IRE1B,* and the rice *OsIRE1* are lethal in standard conditions of growth. In our work, while an *ire1a ire1c* knockout allele was identified, an *ire1b ire1c* homozygous knockout allele was not isolated under standard growth condition. Reciprocal crosses show abnormal segregation of *ire1b* and *ire1c* alleles both using WT or the *ire1b*+/− *ire1c*+/− as pollen source, suggesting that both the male and female gametogenesis is largely impaired when *IRE1C* is knocked out and *IRE1B* is functionally depleted. It has been previously shown that the *ire1a ire1b* mutant is male sterile under heat stress, posing that IRE1 is involved in pollen development in heat stress conditions in Arabidopsis (Deng et al., 2016). Our data support these findings but also extend them by providing evidence that IRE1C is required for the role of IRE1B in gametogenesis. The allele segregation of the *ire1a ire1c* was as expected for a Mendelian segregation ratio, suggesting that IRE1C is involved in gametogenesis independent of IRE1A. Interestingly, only *ire1b*+/− *ire1c*+/+ and *ire1b*+/+ *ire1c*+/− progenies were recovered when either WT or *ire1b*+/− *ire1c*+/− was used as pollen source. No WT progeny was identified when WT was used as pollen source, and only one WT progeny was recovered when *ire1b*+/− *ire1c*+/− was used as pollen source in our analysis, and no *ire1b−/− ire1c−/−* have been recovered in either cases. Such unusual segregation ratio is normally caused by gene linkage effect; however, this is unlikely the case because the *IRE1B* and *IRE1C* loci are located on different chromosomes. Another possibility for the abnormal segregation might be the failure of separation of the WT alleles (*ire1b*+ *ire1c*+) and the homozygous allele (*ire1b*‐ *ire1c*‐), which may be due to defects in meiosis; however, at what stage the segregation of the alleles may be impaired is yet unknown. The recovery of the only WT allele could be due to incomplete penetrance of IRE1C in regulation of gametogenesis or residual functions of IRE1B in the *ire1b* functional knockdown allele. It is yet unclear how IRE1B and IRE1C may be necessary for gametogenesis. One possibility is that in WT the kinase function of IRE1B and IRE1C may phosphorylate partially overlapping substrates. Support to this possibility is provided by the findings that the mammalian IRE1 can phosphorylate other substrates other than itself, at least in vitro (Ali et al., 2011; Feldman et al., 2016). Another possibility is that IRE1B and IRE1C together may form a scaffold for essential proteins, as proposed for non‐plant IRE1 (Chen & Brandizzi, 2013; Woehlbier & Hetz, 2011), and/or their ribonuclease activity may control the mRNA of essential RIDD substrates. A physiological role of RIDD in multiple biological processes has been demonstrated in mammalian cells (Coelho & Domingos, 2014) and may occur also in plants (Bao et al., 2018). Independently from the mechanisms adopted by IRE1C to favor physiological growth, especially reproduction, our results indicate that its function is likely partially overlapping with IRE1B. Besides, our results also showed extremely high expression of IRE1C in the siliques, suggesting a function in seed and silique development, which might also contribute to the abnormal transmission of alleles.

Taken together, the results presented in this study bring to light the existence of a new component of the physiological UPR in Arabidopsis. IRE1C functions in the physiological UPR, specifically in gametogenesis, and operates independently from IRE1A. Nonetheless, IRE1C is essential when IRE1B is depleted posing that the regulation of the physiological UPR in Arabidopsis is much further complex than previously anticipated.

While this manuscript was under revision, complementary work on the characterization of IRE1C was published (Mishiba et al., 2019). The authors indicate that they were not able to isolate an *ire1b ire1c* homozygous knockout allele using the same combination of parental alleles used in our work. Although a quantification of allele segregation in reciprocal crosses was not performed in that work, their publication supports our results for an absolute requirement of IRE1C in conditions of functional downregulation of IRE1B. Our results are also in agreement with their results for a dispensable role of IRE1C in chronic UPR.

## CONFLICT OF INTEREST

The authors declare no conflict of interest.

## AUTHOR'S CONTRIBUTION

Y.P., C.R., and E.R.A. and F.B. designed the experiments. Y.P., C.R., and E.R.A. performed the experiments. Y.P. and F.B. wrote the manuscript.

## ACCESSION NUMBERS

The gene accession numbers that were used in this study are as follows: *UBQ10* (AT4G05320), *ACT2* (AT3G18780), *IRE1A* (AT2G17520), *IRE1B* (AT5G24360), *IRE1C* (AT3G11870), *bZIP60* (AT1G42990), and *BiP3* (AT1G09080).
