# Supplementary material for: AtIRE1C, an unconventional isoform of the UPR master regulator AtIRE1, is functionally associated with AtIRE1B in Arabidopsis gametogenesis
Source: Plant Direct. 2019 Nov 28;3(11):e00187. doi: 10.1002/pld3.187 (PMC6883098; doi:10.1002/pld3.187)
Supplement: Supplementary file 1 [file PLD3-3-e00187-s001.pdf]

Figure S1. Alignment of IRE1A, IRE1B and IRE1C protein sequence

```
Q9C5S2 IRE1A_ARATH          1   MPPRCPFLRHLFFLLLLLSPWIMSPCGGAADDVTYPIVPSSPGRRSILQIRREPPTEPNT     60
Q93VJ2 IRE1B_ARATH          1   MR-GSALLDLI--LFLLVSPLAHSFKGSEISK-FYD--------KSISNQISQSDRESGY     48
Q9SF12 IRE1L_ARATH          1   ------------------------------------------------------------      0

Q9C5S2 IRE1A_ARATH         61   KLVVDRDGKVFLKQQPKETPYWSFSTGSPMHSLYQAPANNNTENATEIT--RPHIIVEY-    117
Q93VJ2 IRE1B_ARATH         49   VLVSTVDGSISLVDMSSQKLDWTFHTNEPIYSSYQAPHYHYTTDEERSSVLGDDFYMDCD    108
Q9SF12 IRE1L_ARATH          1   ------------------------------------------------------------      0

Q9C5S2 IRE1A_ARATH        118   ----LNNSKAATTVDGYHNWTVQEFFRQKPLVTDDGVTLGSETTSAYLVDGRSGRLIHVY    173
Q93VJ2 IRE1B_ARATH        109   KDWRLYNSSVRKGKRVNEIVDASEFIGTLPYTSTDRIVLGKKDTSVFLLDWKTGKLVKRY    168
Q9SF12 IRE1L_ARATH          1   ------------------------------------------------------------      0

Q9C5S2 IRE1A_ARATH        174   KSTGDTKITNALVKPAS--------------------TEDFVNEPLLIRRTDSKLEHFSK    213
Q93VJ2 IRE1B_ARATH        169   RMDELYS--NTVVENDKEKAIVLSKEAPLLFGSGFKKSEDF-PELVYIERKDFKIQCI-S    224
Q9SF12 IRE1L_ARATH          1   ------------------------------------------------------------      0

Q9C5S2 IRE1A_ARATH        214   TTGKLVWNLTVSHFRAALLCDPV--FNSGYD--LGP------------------------    245
Q93VJ2 IRE1B_ARATH        225   KFGDVLWSVSYAKMEAKLQNHESVQFISGLSSSVGKNQFPLSYTTSVPMVQLRNVKYETL    284
Q9SF12 IRE1L_ARATH          1   ------------------------------------------------------------      0

Q9C5S2 IRE1A_ARATH        246   -----KLQTGIYMPLLC----------GSQIDVRGPEIVIRVLHDQPMNVKMLPSPSLNH    290
Q93VJ2 IRE1B_ARATH        285   FPRLGFLDEALYLPFQDRKPNQLAIGDGNQLTLPGNKE--------AEEVLSLPLPETVI    336
Q9SF12 IRE1L_ARATH          1   ------------------------------------------------------------      0

Q9C5S2 IRE1A_ARATH        291   FESENSIMPFGKARESRKLQEQHKQKYTYLFGQWSPVKLLAPLVLLGVVVSVFIKKFSSR    350
Q93VJ2 IRE1B_ARATH        337   SQIT-DI-IDGSTK-QAGFASKFSGLIVLIFGFCVTMLSVCGLFFYRLRQSIRIKE--PY    391
Q9SF12 IRE1L_ARATH          1   --------------------------------MWLLAISLVGLLVVVVCVFLRFSK--DK     26
                                                                       :  *..  :   : :.:

Q9C5S2 IRE1A_ARATH        351   GSDVSLKAGPSKKKKNRK-------SAKDTNRQ-SVPR--------G-QDQFELIEG---    390
Q93VJ2 IRE1B_ARATH        392   VSEVPI-ATPKKK----K-------SKKNGTTK-AVHKKENGFISGGNKDPSHEENE---    435
Q9SF12 IRE1L_ARATH         27   GLD----GIVNEKKRDKNSAPRVSASGEDGTKNEQVEKKS--DPSGGLGEENEKTNSESK     80
                                  :    .  .:*    :       * :: . :  * :        *  :  .  :
```


```
Q9C5S2 IRE1A_ARATH        351   GSDVSLKAGPSKKKKNRK-------SAKDTNRQ-SVPR--------G-QDQFELIEG---    390
Q93VJ2 IRE1B_ARATH        392   VSEVPI-ATPKKK----K-------SKKNGTTK-AVHKKENGFISGGNKDPSHEENE---    435
Q9SF12 IRE1L_ARATH         27   GLD----GIVNEKKRDKNSAPRVSASGEDGTKNEQVEKKS--DPSGGLGEENEKTNSESK     80
                                  :    .  .:*    :       * :: . :  * :        *  :  .  :
```


```
Q9C5S2 IRE1A_ARATH        391   -----------GQMLLGF-----NNFQSGATDGRK-IGKLFLSSKEIAKGSNGTVVFEGI    433
Q93VJ2 IRE1B_ARATH        436   -----------KRLLTAF-----PGLNNSSAEGYR-VGKLFVSNKEIAKGSNGTVVLEGS    478
Q9SF12 IRE1L_ARATH         81   VLSVPSDQNINKTLPVMLPSLELRKYDENETPGKVVNRRLLVSTNEMKYGRNGYEVFQGV    140
                                             :   :        :.. : *     :*::*.:*:  * **  *::*

Q9C5S2 IRE1A_ARATH        434   YEGRPVA-VKRLV--RSHHEVAFKEIQNLIASDQHTNIIRWYGVEYDQDFVYLSLERCTC    490
Q93VJ2 IRE1B_ARATH        479   YEGRLVA-VKRLV--QSHHDVAQKEILNLMASDKHSNIVRWYGVDQDEHFIYISLELCAC    535
Q9SF12 IRE1L_ARATH        141   YGRRSSVAVKCLDLAHTTEAFIQNEIDNHCLCDDHSNIIRFHGLEQDQSFAYICLEPWKC    200
                                *  *  . ** *   :: . .  :** *   .*.*:**:*::*:: *: * *:.**   *

Q9C5S2 IRE1A_ARATH        491   SLDDLIKSYLEFSMTKVLENNDSTEGVAAYKIQLDSLEGVIKGNNFWKVGGHPSPL--ML    548
Q93VJ2 IRE1B_ARATH        536   SLNDLIYASSALL-----ESPMA--SSSIHSIQINPIFENGKGVELWKENGHPSPV--LL    586
Q9SF12 IRE1L_ARATH        201   SLDDLIKLSVRRTK-----------RDTQAVAPVDDLEKVMKRIKFWKEKGKPLPLTPML    249
                                **:***                     :     :: :    *  ::**  *:* *:  :*

Q9C5S2 IRE1A_ARATH        549   KLMRDIVCGIVHLHELGIVHRDLKPQNVLI-SKDMTLSAKLSDMGISKRMSRDMSSLGHL    607
Q93VJ2 IRE1B_ARATH        587   KLMRDIVAGLVHLHDIGIVHRDLKPQNVLI-VKNSSLCAKLSDMGISKRLPADTSALTRN    645
Q9SF12 IRE1L_ARATH        250   KLMRDVVCGLAHLHKLKTIHRNLNPQNVLIIVKDMTLTAKISDMSLSKHLGGKKSSYKHL    309
                                *****:*.*:.***.:  :**:*:******  *: :* **:***.:**::  . *:  :

Q9C5S2 IRE1A_ARATH        608   --ATGSGSSGWQAPEQLLQGRQT---RAVDMFSLGCVIFYTITGCKHPFGDDLERDVNIV    662
Q93VJ2 IRE1B_ARATH        646   STGLGSGSSGWQAPEQLRNERQT---RAVDLFSLGCVLFFCMTGGKHPYGDNYERDVNVL    702
Q9SF12 IRE1L_ARATH        310   --ATCSGSSGWQAPEQLNKDKKKKEDFPADMFNFGCLLHYAVMG-THPFGSPSERDTNIK    366
                                  .  ************ : ::.     .*:*.:**::.: : * .**:*.  ***.*:

Q9C5S2 IRE1A_ARATH        663   KNKVDLFLVEHVPEASDLISRLLNPDPDLRPSATEVLLHPMFWNSEMRLSFLRDASDRVE    722
Q93VJ2 IRE1B_ARATH        703   NDQKDLFLIESLPEAVHLLTGLLNPDPNLRPRAQDVMHHPLFWNSDMRLSFLRDASDRVE    762
Q9SF12 IRE1L_ARATH        367   TNNKTNLSLVTNLEAINLIEQLLNYKPDLRPSATQVLLHPLFWDSEKRLFFLREASDRIE    426
                                .::   : :    ** .*:  *** .*:*** * :*: **:**:*: ** ***:****:*

Q9C5S2 IRE1A_ARATH        723   LENREADSEILKAMESTAPVAIGGKWDEKLEPVFITNIGR-------------YRRYKYD    769
Q93VJ2 IRE1B_ARATH        763   LENREEGSQLLAALESTAAVTLNGRWDEKLDSIFLDNIGR-------------YRRYKFD    809
Q9SF12 IRE1L_ARATH        427   LDITMWGDLN--KTIAPRVLGESKDWASKLGKTFITHIENLAQAQPGQESRQYNRSYKYW    484
                                *:    ..       :   :  .  * .**   *: :* .              * **:

Q9C5S2 IRE1A_ARATH        770   SIRDLLRVIRNKLNHHRELP--PEIQELVGTVPEGFDEYFAVRFPKLLIEVYRVISLHCR    827
Q93VJ2 IRE1B_ARATH        810   SIRDLLRVIRNKLNHYRELP--KELQELLGSVPEGFERYFSSRFPKLLIQVYTVLFDYCN    867
Q9SF12 IRE1L_ARATH        485   SLRHLLRLIRNILSHHREILDDPKIKEMVGKVPEGLDIFFTARFPNLMMEIYAFISMHCK    544
                                *:*.***:*** *.*:**:    :::*::*.****:: :*: ***:*::::* .:  :*.

Q9C5S2 IRE1A_ARATH        828   EEEVFRKYFKCDII                                                  841
Q93VJ2 IRE1B_ARATH        868   NEEFFFKYSKTTVF                                                  881
Q9SF12 IRE1L_ARATH        545   GEEAFEKYFN----                                                  554
                                 ** * ** :
```

Signal peptide
Transmembrane domain
Ribonuclease domain
Kinase domain

Figure S2. Localization of IRE1C-YFP and ER-ck in tobacco leaves

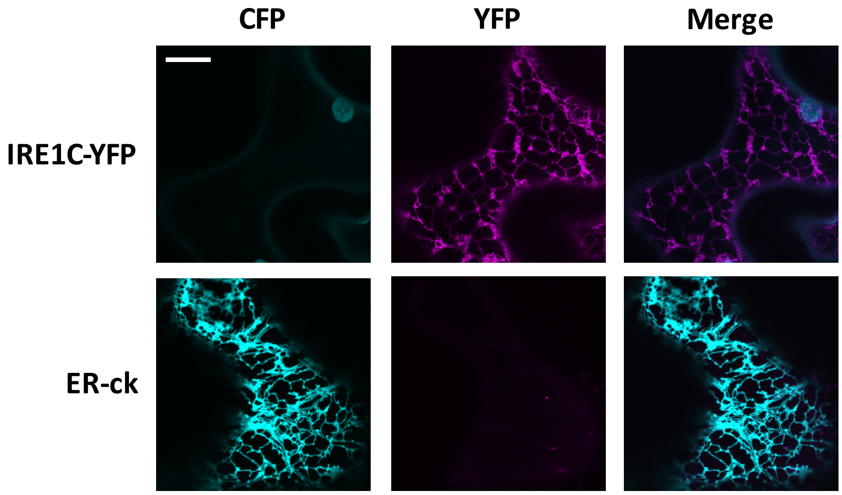

Figure S3. Genotyping of reciprocal crosses progenies

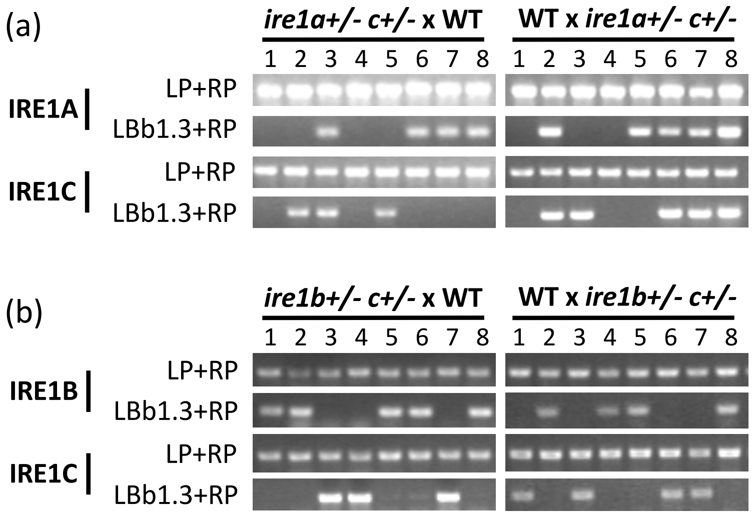

Figure S4. *IRE1C* expression in different tissues or organs

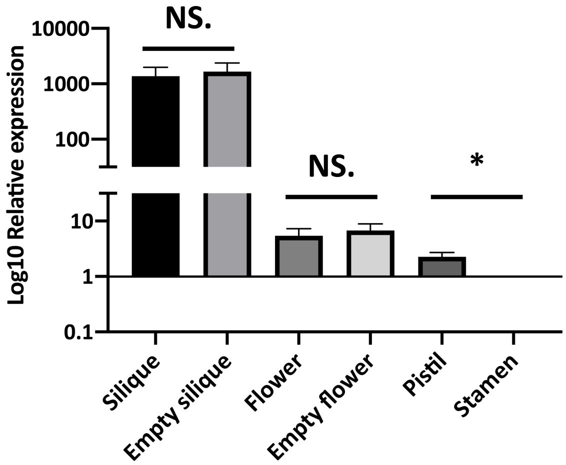

Figure S5. Phylogenetic tree of *IRE1C*

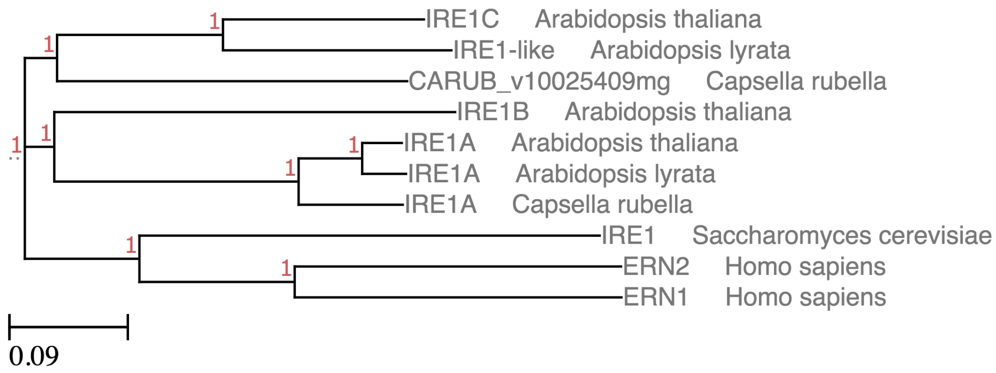

Table S1. Primers used in this study

Primer name	Primer sequence (5' – 3')	Purpose
UBQ10-F	TCAATTCTCTCTACCGTGATCAAGATGCA	RT-PCR of *UBQ10*
UBQ10-R	GGTGTCAGAACTCTCCACCTCAAGAGTA	
IRE1A-F	AGACCCTGATTTACGTCCTAGC	RT-PCR of *IRE1A*
IRE1A-R	CCGACAAGTTCCTGAATTTCCG	
IRE1B-F	CAAATTTGAGACCGAGAGCAC	RT-PCR of *IRE1B*
IRE1B-R	CTAGAATACAGTGGTCTTAG	
IRE1C-F	TAAGCAAGCATCTCGGTGGG	RT-PCR of *IRE1C*
IRE1C-R	CCTGAGTTGCACTAGGACGTA	
WiscDsLox420D09-LP	CAAAATCTTCAGTGCTAGCGG	Genotyping of *ire1a*
WiscDsLox420D09-RP	TATCTCCGATCCATCGTTGAC	
WiscDsLox-LP	AACGTCCGCAATGTGTTATTAAGTTG	
SAIL_238_F07LP	CCTCTCGAACCCTTCAGGTAC	Genotyping of *ire1b*
SAIL_238_F07RP	GAAGGAAAACGGACATCCTTC	
SAIL-LB2	GCTTCCTATTATATCTTCCCAAATTACCAATACA	
SALK_204405-LP	CAAACAAACACAACAACACGG	Genotyping of *ire1c*
SALK_204405-RP	TGTGGTTATTGGCCATCTCTC	
LBb1.3	ATTTTGCCGATTTCGGAAC	
IRE1C_RT-F	GACGAGAACGAAACCCCTGG	RT-PCR of *IRE1C*
IRE1C_RT-R	GAACTACCAGAACATGTTGCAAGGT	
IRE1Cgeno-F	ATGTGGTTATTGGCCATCTCTCTTGT	Cloning of IRE1C genomic DNA
IRE1Cgeno-R	TGGCGTTGAAGTACTTCTCGAACGCT	
IRE1A_qRT-F	GGGAATAGTTCATCGGGACTTG	qRT-PCR of *IRE1A*
IRE1A_qRT-R	CTCTGGACATACGCTTGCTAAT	
IRE1B_qRT-F	GGTGGGATGAGAAACTGGATAG	qRT-PCR of *IRE1B*
IRE1B_qRT-R	TCCGTATGACCCGTAACAAATC	
IRE1C_qRT-F	GGAGCCTTCGCCACTTATT	qRT-PCR of *IRE1C*
IRE1C_qRT-R	AGGAACCTTTCCCACCATTT	
ACT2-F	GGAAGGATCTGTACGGTAAC	RT-PCR of *ACT2*
ACT2-R	GGACCTGCCTCATCATACT	
