# Supplementary material for: AtIRE1C, an unconventional isoform of the UPR master regulator AtIRE1, is functionally associated with AtIRE1B in Arabidopsis gametogenesis
Source: Plant Direct. 2019 Nov 28;3(11):e00187. doi: 10.1002/pld3.187 (PMC6883098; doi:10.1002/pld3.187)
Supplement: Supplementary file 2 [file PLD3-3-e00187-s002.pdf]

Dear Editors,

Thank you for the positive assessment of our work. We sincerely appreciate the suggestions of the reviewers. As you will see in the detailed response letter to the reviewers, we have attended all their requests by amending the figures and edited the manuscript text. Specifically, we have added citation of qPCR for bZIP60 spliced variants for Reviewer #1's suggestion. We have also added more explanation for the non-Mendelian segregation pattern for Reviewer #2. We have also completed the statistical analysis in the original Figure 2, as suggested by both reviewers. The detailed response to each comment from the reviewers is attached in the "response to reviewers letter.

We hope the revised manuscript will be considered suitable for publication.

Sincerely,

Federica Brandizzi

RE: AtIRE1C, an unconventional isoform of the UPR master regulator AtIRE1, is functionally associated with AtIRE1B in Arabidopsis gametogenesis

Dear Dr. Brandizzi:

Thank you for submitting to Plant Direct. All required reviews have been returned and we have now finished our evaluation of your manuscript. In light of the reviewers' and editor's comments, further revisions are needed before the paper can be accepted for publication in Plant Direct.

Please revise your manuscript in accord with the reviewers' comments (pasted below). The specific concerns that need to be addressed to meet the Plant Direct criteria are: Reviewer #1 suggests to provide detailed information about how to differentiate splice variants of bZIP60 in qPCR. Figure 2b did not match with its legend. Reviewer #2 was concerned about Non-Mendelian segregation pattern in Table 1. Both reviewers pointed out that statistical analysis in some assays were missing, e.g. Figure 2e and 2f. If these concerns can be fully addressed, we would not send the revised manuscript back for another round of review. The reviewers also made several other helpful suggestions.

When uploading the revised version of this article, please be sure to include the following:

-A word document that contains your response to the reviewers. You should respond to each reviewer comment and note the changes made to the manuscript. If you do not agree with a reviewer's comment and choose not to make a suggested revision, please explain why. Please try to provide as complete an answer as possible to each reviewer's criticisms.
-A tracked changes document with each change highlighted
- A clean version of the latest version of the manuscript

To upload your revision, please click the link below.
https://plantdirect.msubmit.net/cgi-bin/main.plex?el=A7Lr5IF2A2CLs3I1A9ftdd9keciRzhVEkfT6UdPaLQAZ

In order to provide as timely a service as possible, we ask that your revision is resubmitted within three months after receipt of this request. If an extension is needed, please send a request, along with a brief explanation, to the editorial office at plantdirect@wiley.com .

Please note that, in addition to publishing reviewer comments, the author's responses to review comments will also be published alongside the final version of the paper. If you would not like the author's responses to be published, please contact the editorial office at plantdirect@wiley.com .

Thank you very much for giving us an opportunity to review your work. I look forward to receiving the next version.

Sincerely,

Ying Gu

Hsou-min Li

Editor, Plant Direct

-- Reviewer comments:

Reviewer #1:

This manuscript characterizes a novel IRE1 isoform in Arabidopsis. IRE1C which lacks the lumenal sensor domain present in other IRE1 proteins, is demonstrated to have a role in gametogenesis, and a potential role in the UPR. Overall this work was well conceived and carried out, and the data support the conclusions. However, there are some minor issues that should be addressed.

1. Please provide more information about how the spliced bZIP60 was differentiated from the unspliced version in qPCR.

We have used specific primers for the spliced and unspliced forms that we used in earlier work (Moreno et al. 2012. PLoS One). We added the information and the citation. Thank you for raising this point.

2. In the discussion, the authors claim that this isoform exists only in Arabidopsis. Please provide data or citation to support this claim.

We appreciate the reviewer's comment about this. To confirm our claim, we performed the phylogenetic analysis on IRE1C with more stringent parameters, and we found a potential IRE1C isoform in Capsella rubella. We have added a phylogenetic tree in the supplemental figures, and have edited the text in discussion.

3. Please provide quantification and statistical analysis of the aborted pollen phenotype.

Done. Thank for the suggestion,

4. Figure legends refer to "histograms", these are not histograms they are bar or column graphs. Please correct.

You are correct. Done.

5. An earlier paper, (not referenced in manuscript; Moreno, Mukhtar, et al., 2012 Plos One) showed that ire1a and ire1b single mutants were more sensitive to ER stress than WT. Please address this discrepancy. Please also indicate which alleles were used in the current study.

The alleles used in this study are listed in the Experimental Procedures section. As for the single mutant sensitivity, we think it may due to the different experimental conditions used to induce the UPR in our study and the Moreno 2012 paper. In their work, Moreno et al. used MS media containing 0.3 µg/mL Tm to test the survival of seedlings. In our work, we used half-strength LS media with 25 nM Tm for chronic ER stress treatment, and 0.5 µg/mL Tm in liquid half-strength LS media for temporary ER stress treatment. We were not able to find the light intensity parameters in Moreno et al., 2012, which could be different and likely influencing the UPR. We have included the light regime used for our experiments in the manuscript.

6. The manuscript needs careful proofreading.

Agree. Done.

Reviewer #2:

The manuscript about AtIRE1C by Pu et al. described the function study of the AtIRE1C gene. As the title suggested, the authors drew the conclusion that AtIRE1C is "an unconventional isoform of the UPR master regulator 1 AtIRE1" and "functionally associated with AtIRE1B in Arabidopsis gametogenesis". While the authors did show some interesting results such as impaired seed development in heterozygous mutants, the experimental results presented in this manuscript are questionable and cannot support their claim.

My biggest concern or confusion is the absence of WT in the progenies of some of the crosses (Table 1). For instance, when +/b+/c was pollenated by itself or WT pollens, its progenies have

no WT. It is indeed intriguing, but the authors did not explain clearly why this happened. In classic genetics, this usually indicates those two genes/alleles may not separate independently during meiosis or they are located very close to each other. However, this should not be the case: ireb and irec were located on different chromosomes. This led the inspection and suspicion on how the genotyping was done, especially for irec mutant. The Salk_204405 was used as the irec knockout. Without clear description of which exact primers were used for identifying the insertion and WT, it is hard to judge if the PCR was set up correctly for genotyping. With the limited information provided in Table S1 (two SALK_204405-LP primers), my suspicion/explanation would be the authors did not use a T-DNA specific primer? That might be a bold guess of what happened.

We apologize for the typo on the primer list. Primers used for genotyping including the LB primers have been added to the list, and the use for each primers have also been added to the list. We have also added supplemental Figure S3 as example of our genotyping. Given the controls included in the work, we do not believe there are issues with our genotyping method, and that the abnormal segregation is due to meiosis defects.

Other comments:
Line 161-162, "as detailed below", there is no detail about T-DNA indetification.

Text has been edited for clarity.

Line 177, most references are mentioned as this line where two authors "(Meng, Ruberti et al. 2017)" were mentioned, a new style?

We've edited the reference style.

Line 192, Table S1, primer sets are not clear.

Amended to make it clear. Thank you.

Line 239-243, it would be better to show the sequence alignment instead of percentage of identical AA residue.

Done.

Line 290-320, the authors presented the impaired seed development phenotype of ire1a+/- ire1b+/- ire1c+/- mutant but not showing that of ire1b+/- ire1c+/-; the results of reciprocal crosses were done for ire1b+/- ire1c+/- but not for ire1a+/- ire1b+/- ire1c+/-. A thorough investigations should include both experiments for both heterozygous mutants.

Done. We have included the ire1b+/- ire1c+/- mutant in our analyses. Both 1a+/-1b+/- 1c+/- and 1b+/- 1c+/- mutants have been tested for the siliques and pollen phenotypes as in Figure 2. Thank you for the suggestion.

Line 296-298, the authors claimed the "unfertilized ovules", yet Figure 2e does not necessarily indicate "unfertilized ovules", it could show failed fertilized ovules.

We've edited the text for clarity.

Line 312-315, the results are odd, not explained well.

We've added more details such as the expected segregation ratio in the text, and we have also expanded Table 1 for clarity. We think this suggestion was particulalry helpful. Thank you.

Line 388-389, the statement of "nearly no expression IREB" seemed a little conflicting with the lethal phenotype of complete loss of IREB (line 104-105). The ireb mutant used in this study is probably more of a knockdown instead of a knockout.

Yes, we indicated that our ire1b mutant is a functional knockdown. As discussed in an earlier publication (Chen and Brandizzi 2012, The Plant Journal), the allele used in this work may lack a functional RNAse domain.

Line 463-464, the authors concluded that IREC does not play a role in roots, why not investigate the expression pattern in other tissues/organs? Especially in siliques/seeds, or gametophyte as the authors suggested where IREC plays a critical role when IRECB is compromised.

Done. qRT-PCR results of IRE1C expression in different tissues/organs are shown in Figure S4.

Line 479-484, the explanation of the absence of WT is very vague, not convincing.

We have expanded the text in the discussion for better explanation.

From Figure 1f, the authors may get more information by investigating the IREC expression and function in silique/seed development.

Done. See Figure S4.

Any statistical analysis for Figure 2f?

Done.

Table S1 has two SALK_204405-LP primers. The authors should be more clear about how those primers were used. There is no T-DNA primer listed?

We apologize for our mistakes. The table has been updated.
